# Fructose-induced FGF21 secretion does not activate brown adipose tissue in Japanese young men: randomized cross-over and randomized controlled trials

**DOI:** 10.1186/s40101-023-00353-0

**Published:** 2024-01-04

**Authors:** Haruki Kataoka, Shinsuke Nirengi, Yuka Matsui, Hirokazu Taniguchi

**Affiliations:** 1 Division of Applied Life Sciences, Graduate School of Life and Environmental Sciences, Kyoto Prefectural University, Kyoto, Japan; 2 Clinical Research Institute, Division of Preventive Medicine, National Hospital Organization Kyoto Medical Center, Kyoto, Japan; 3 Dorothy M. Davis Heart and Lung Research Institute, Department of Physiology and Cell Biology, The Ohio State University Wexner Medical Center, Columbus, OH USA

**Keywords:** Brown adipose tissue, Fibroblast growth factor 21, Thermogenesis, Fructose, Thermography, RCT

## Abstract

**Background:**

Human brown adipose tissue (BAT) activity is associated with lower body fatness and favorable glucose metabolism. Previous studies reported that oral fructose loading induces postprandial fibroblast growth factor 21 (FGF21) secretion. FGF21 is a known inducer of adipose tissue thermogenesis; however, the effects of diet-induced FGF21 secretion on BAT thermogenesis remain to be elucidated.

**Methods:**

The effects of both single load and daily consumption of fructose on BAT activity were examined using a randomized cross-over trial and a 2-week randomized controlled trial (RCT), respectively. In the cross-over trial, 15 young men consumed a single dose of fructose solution or water and then consumed the other on a subsequent day. The RCT enrolled 22 young men, and the participants were allocated to a group that consumed fructose and a group that consumed water daily for 2 weeks. BAT activity was analyzed using thermography with cold exposure. Plasma FGF21 level was determined by enzyme-linked immunosorbent assay.

**Results:**

In the cross-over single-load trial, plasma FGF21 levels were significantly increased at 2 h after oral fructose load (*p* < 0.01); however, there was no significant difference in BAT activity between the fructose load and drinking water. The 2-week RCT revealed that both plasma FGF21 levels and BAT activity were not significantly increased by daily fructose consumption compared to water. Correlation analyses revealed that BAT activity at the baseline and the final measurements were strongly and positively associated with the RCT (*r* = 0.869, *p* < 0.001). Changes in BAT activity were significantly and negatively correlated with changes in plasma glucose levels during the 2-week intervention (*r* = − 0.497, *p* = 0.022).

**Conclusions:**

Oral fructose load induces a temporary increase in circulating FGF21 levels; however, this does not activate BAT thermogenesis in healthy young men. Further studies are needed to elucidate the effect of endogenous FGF21 on physiological function.

**Trial registration:**

This study is registered with the University Hospital Medical Information Network in Japan (number 000051761, registered 1 August 2023, retrospectively registered, https://center6.umin.ac.jp/cgi-open-bin/ctr/ctr_view.cgi?recptno=R000052680).

## Background

Adipose tissues have different functional features. White adipose tissue is a regulator of energy storage and systemic metabolic homeostasis [1], whereas brown adipose tissue (BAT) converts metabolic energy into heat and thereby increases energy expenditure [2, 3]. In adult humans, the BAT depot is mainly localized in the supraclavicular region, and the heat production of BAT is activated by cold exposure [2, 3]. The cold-induced activation of BAT (BAT activity) induces energy expenditure and glucose disposal [4–6]. High levels of BAT activity are associated with lower body fatness [7, 8] and favorable glucose metabolism [9, 10]. Interventional studies reported that BAT activity was increased by oral intake of food ingredients such as capsinoids [4, 11] and tea catechins [12, 13]. Thus, BAT activity has health benefits, and the activity could be induced by environmental and/or nutritional factors.

Fibroblast growth factor 21 (FGF21) is known as an endocrine and metabolic regulator that induces adipose tissue thermogenesis [14, 15]. FGF21 administration prevents body weight gain and the development of an insulin-resistant state in rodents [16]. Consistent results were shown in human studies, which reported that administration of FGF21 analogs decreased body weight and increased insulin sensitivity in people with type 2 diabetes [17, 18]. Other human studies suggested that blood FGF21 levels were significantly and positively associated with BAT activity [19, 20]. Therefore, endogenous secretion of FGF21 may have a physiological role in human energy metabolism. Recent studies reported that the secretion of FGF21 was stimulated by acute oral fructose load [21–23], which secreted hepatic FGF21 in a carbohydrate response element binding protein (ChREBP)-dependent manner [24]. The results suggest that the FGF21 response to fructose intake may activate BAT thermogenesis. On the other hand, Richard et al. reported that a short-term high-fructose diet suppressed glucose uptake by cold-stimulated BAT in young men [25], suggesting the adverse effects of fructose consumption on BAT activity.

As the effects of diet-induced secretion of FGF21 on BAT thermogenesis activation remain to be elucidated, the present study aimed to evaluate the acute and short-term effects of fructose loading on BAT activity using a randomized cross-over trial and a randomized controlled trial (RCT).

## Methods

### Ethics approval and trial registration

The present study was approved by the Ethical Committee of Kyoto Prefectural University (approval number 216). All participants provided written informed consent before enrolment. The study is registered with the University Hospital Medical Information Network in Japan (number 000051761) and was conducted in accordance with the Declaration of Helsinki.

### Study design and participants

**Fig. 1:**
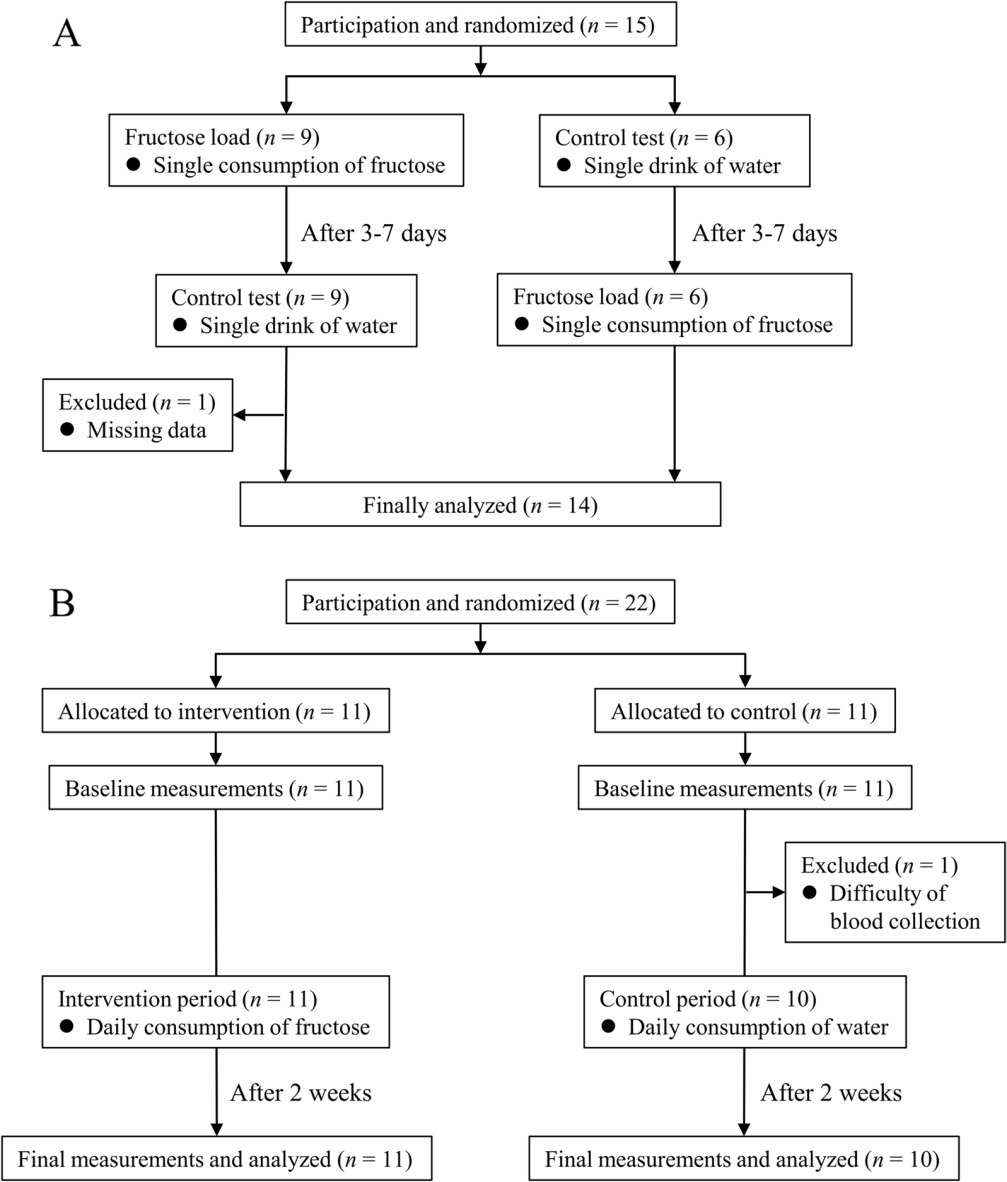
Flow diagram of the participants in the study. **A** Randomized cross-over trial for single fructose load. **B** Randomized controlled trial consisting of daily fructose consumption for 2 weeks

The effects of both single load and daily consumption of fructose on BAT activity were examined in the winter of 2022 (Fig. 1) by randomized cross-over trial and RCT, respectively. Participants were healthy young men aged over 18 years.

In the cross-over trial (Fig. 1A), 15 young men (aged 20–24 years) participated and were randomly divided into a group that received a single load of fructose (*n* = 9) and a group that received water (*n* = 6). The subjects switched to the other group after 3–7 days of the first dose. The data of one participant who did not complete the study was excluded; thus, the data of 14 participants were finally analyzed.

The RCT included 22 young men aged 18–24 years (Fig. 1B). The participants were randomized and allocated to a group that consumed fructose (*n* = 11) and a group that consumed water (*n* = 11). After baseline measurements, one participant in the group that consumed water declined to participate due to syncopal reaction after blood collection, and their data was excluded. The remaining 21 participants completed the 2-week intervention, and final measurements were performed.

### Fructose intake

A fructose solution was prepared by dissolving 30 g of fructose in 225 mL of a commercial brand of water, as described in a previous study that examined a quantitative relationship between fructose load and FGF21 secretion [23]. The fructose solution was used in both the cross-over and controlled trials. A commercial brand of water was used as a control.

### Measurement of BAT activity

BAT activity was determined using mild cold exposure, as described previously [20, 26]. In brief, subjects were seated at rest in a temperature-controlled room at 27 °C for 30 min. Baseline body surface temperature was measured using a thermal imaging camera (DETC1000T; D-eyes, Osaka, Japan). The subjects entered the climatic chamber (TBRR-9A4GX; ESPEC, Osaka, Japan), which was set at 19 °C. After the start of cold exposure, body surface temperature was collected at 30 min and 1 h of mild-cold exposure. Before and during cold exposure, subjects were asked to rate their shivering, cold sensation, and discomfort using a visual analog scale [20, 26].

The supraclavicular temperature adjacent to the BAT location on both the right and left sides was measured from each image. Chest temperature was simultaneously measured as a control for underlying BAT depots. The images of body surface temperature were analyzed using a modified (D-eyes) version of the Thermal-Cam v.1.1.0.9 software (Laon People, Seoul, Korea), and the average of supraclavicular temperature minus chest temperature was estimated as BAT activity. For the 2-week RCT, maximal BAT activity was defined as the higher BAT activity between 30 min and 1 h after cold exposure.

### Anthropometric characteristics, dietary food intake, and physical activity

Body weight and body fat percentage were measured using an electronic scale (V-body HBF-359; Omron, Kyoto, Japan). Body mass index was calculated as body weight (kg) divided by the square of height (m). Energy, protein, carbohydrate, and fat intake were also assessed using a Food Frequency Questionnaire based on the food groups (Ver. 6.0; Kenpakusha, Tokyo, Japan) [27]. Physical activity was estimated by the International Physical Activity Questionnaire and expressed as metabolic equivalents per hour per week [28].

### Measurements in the randomized cross-over trial and RCT

For all measurement days, subjects were instructed to arrive by 9:00 AM after an overnight fast, and the measurements were performed within 3 h.

In the cross-over trial (single load), baseline body surface temperature and blood sample were collected, and then the subjects consumed fructose solution or control water. The initiation of cold exposure was started 1.5 h after the oral fructose intake to match BAT thermogenesis [26] and a peak increase in blood FGF21 level 2 h after fructose load [21–23]. Body surface temperature and blood sample were collected at 30 min and 1 h of cold exposure (2 h and 2.5 h after the oral fructose load, respectively).

In the RCT (daily consumption), the same baseline and final measurements were performed before and after the 2-week daily consumption of fructose solution. Body surface temperature was measured at baseline (before cold exposure) and after 30 min and 1 h of cold exposure. Blood sampling was performed before cold exposure. The subjects did not consume fructose solution and could only consume water in the baseline and final measurements.

### Blood analysis

The collected blood samples were centrifuged at 3000×*g* for 5 min. Plasma and serum were stored at – 80 °C until the time of analysis. The concentrations of plasma glucose and free fatty acid (FFA) were measured using Glucose C2-test Wako and NEFA C-test Wako (FUJIFILM Wako Pure Chemical, Osaka, Japan), respectively. Serum triglyceride, total cholesterol, high-density lipoprotein cholesterol, and low-density lipoprotein cholesterol were determined by Kyoto Biken Laboratories, Inc. (Kyoto, Japan). Plasma FGF21 concentration was determined using a commercially available enzyme-linked immunosorbent assay kit (DF2100; R&D Systems, Minneapolis, USA) according to the manufacturer’s instructions.

### Statistics

All statistical analyses were performed using SPSS, version 29.0 (SPSS, Chicago, IL, USA). The Kolmogorov-Smirnov test was performed to assess the normality of data distribution. Two-way ANOVA analysis (time × condition) and post-hoc Tukey’s HSD test were used to determine the absolute value difference in the cross-over trial. Baseline characteristics and changes in variables during the RCT were compared using Student’s *t* test for normally distributed data or the Mann-Whitney *U* test for nonnormally distributed data. Relationships between changes in all variables were determined by Pearson’s correlation coefficients. All measurements and calculated values are presented as the mean ± SD, and the level of statistical significance was set at *p* < 0.05.

## Results

### Effects of single fructose load on FGF21 and BAT in the cross-over trial

**Fig. 2:**
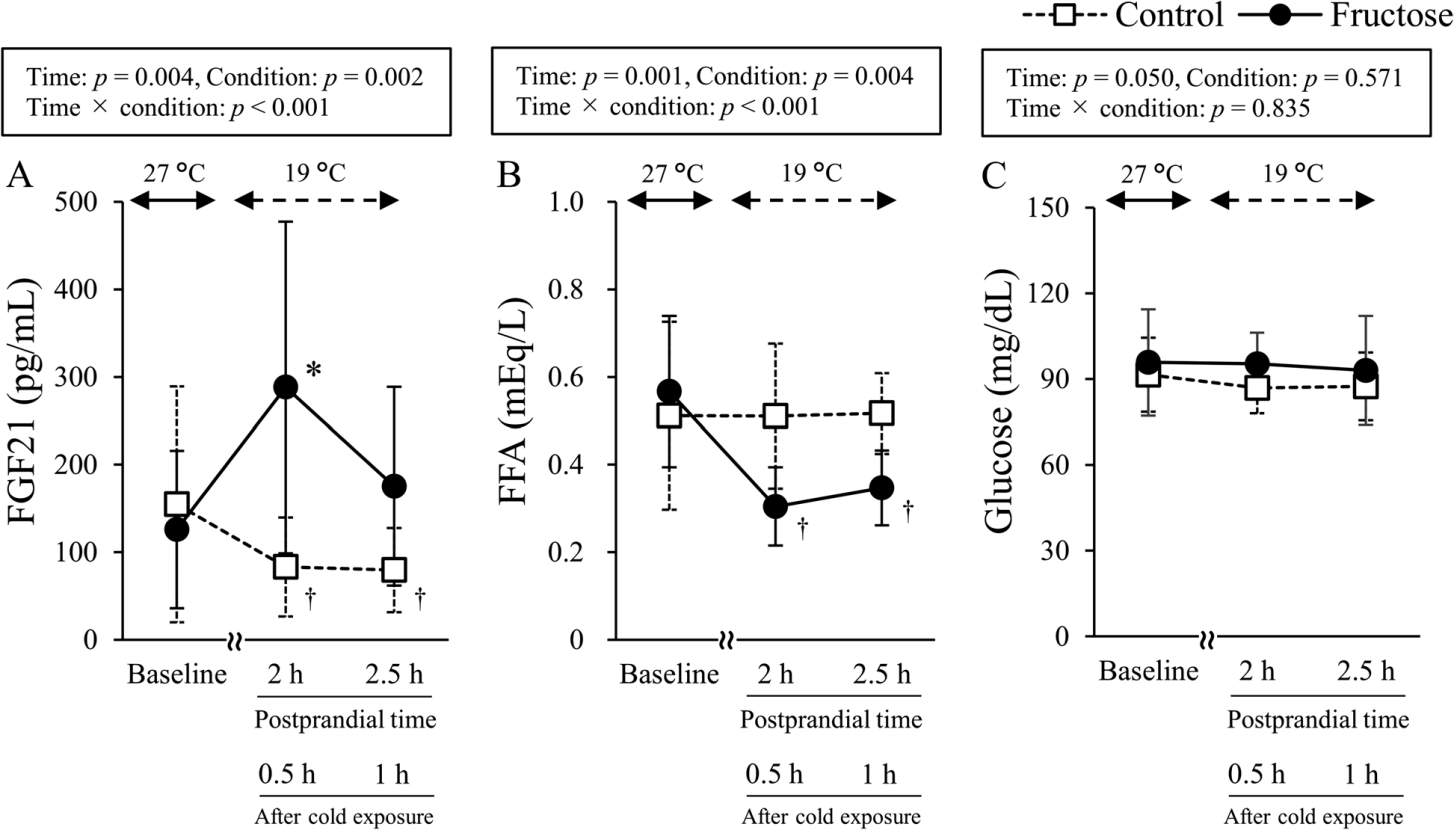
Changes in plasma FGF21, FFA, and glucose levels in the randomized cross-over trial. **A** Plasma FGF21 levels. **B** Plasma FFA levels. **C** Plasma glucose levels. Two-way ANOVA analysis and the post-hoc Tukey’s HSD test were performed. * and ^†^ indicate statistical significance compared to the baseline levels (*p* < 0.05). FGF21, fibroblast growth factor 21; FFA, free fatty acid

**Fig. 3:**
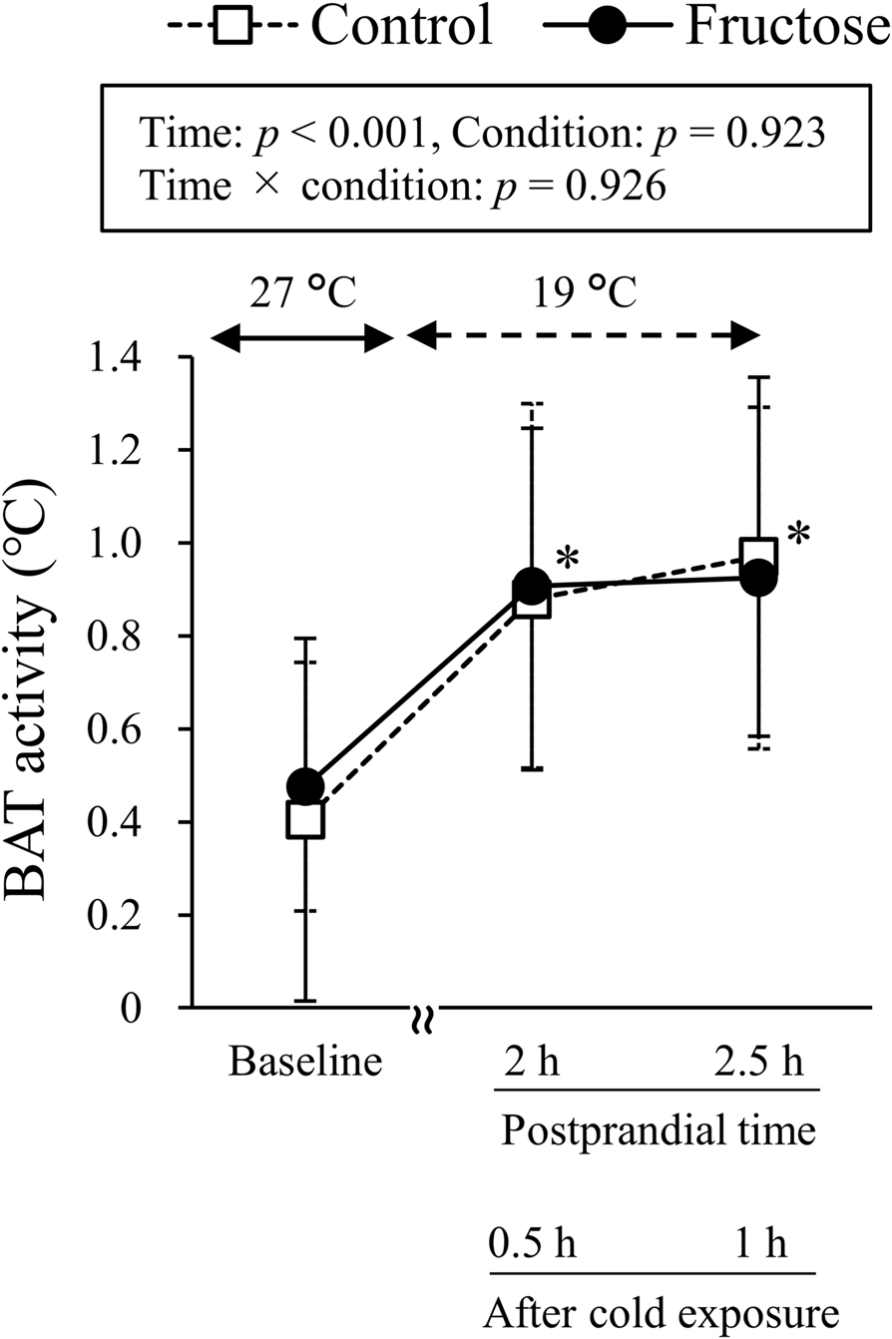
Changes in BAT activity in the randomized cross-over trial. Two-way ANOVA analysis and the post-hoc Tukey’s HSD test were performed. * indicates statistical significance compared to baseline levels (*p* < 0.05). BAT, brown adipose tissue

Subject characteristics for the cross-over single-load trial are shown in Table 1. Shivering response, cold sensation, and discomfort during cold exposure were not statistically different between the fructose load and drinking water conditions (data not shown). Compared to the baseline level, plasma FGF21 levels were significantly increased at 2 h after oral fructose load (*p* < 0.01), but decreased to non-significant levels at 2.5 h after loading (Fig. 2A). Plasma FGF21 levels at both 2 h and 2.5 h after drinking water were significantly lower than at the baseline level (*p* < 0.05). Plasma FFA levels were significantly decreased at both 2 h and 2.5 h after fructose loading (*p* < 0.05) in the group compared to the baseline level (Fig. 2B). There were no significant changes in plasma glucose levels during the single-load trial (Fig. 2C). As shown in Fig. 3, BAT activity, which was evaluated as standardized supraclavicular temperature, was significantly increased by 30-min and 1-h mild cold exposure at both 2 h and 2.5 h after fructose loading, respectively (*p* < 0.01). However, a significant increase in BAT activity was similarly observed after drinking water (*p* < 0.01). Two-way ANOVA analysis found that there was no significant difference in BAT activity between the fructose load and drinking water conditions (*p* = 0.923).

**Table 1:** Subject characteristics for the cross-over single-consumption trial (*n* = 14)

Age (year)	21.8 ± 1.1
Height (cm)	173.1 ± 5.4
Weight (kg)	61.9 ± 7.6
BMI (kg/m^2^)	20.6 ± 2.2
Body fat (%)	15.7 ± 3.5
Blood metabolic parameters	
Glucose (mg/dL)	93.7 ± 13.0
FFA (mEq/L)	0.54 ± 0.15
Triglyceride (mg/dL)	71.8 ± 33.3
Total cholesterol (mg/dL)	180.4 ± 23.6
HDL cholesterol (mg/dL)	65.2 ± 11.9
LDL cholesterol (mg/dL)	98.2 ± 19.9
Dietary consumption (/day)	
Energy (kcal)	1767 ± 435
Protein (g)	60.2 ± 15.5
Fat (g)	64.4 ± 18.3
Carbohydrate (g)	221.6 ± 54.9
PA (METs-h/week)	52.9 ± 38.4

*BMI* body mass index, *FFA* free fatty acid, *HDL* high-density lipoprotein, *LDL* low-density lipoprotein, *MET* metabolic equivalent, *PA* physical activity

### Effects of daily fructose consumption on FGF21 and BAT in the RCT

Table 2 shows anthropometric values, blood metabolic parameters, dietary consumption, physical activity, and BAT activity before and after the 2-week RCT. There were no significant changes in body weight and body fat percentage between the groups. The mean value of plasma glucose levels was reduced in the fructose group, but the difference was not significant (*p* = 0.173). Changes in blood lipid parameters were not different between the groups. Plasma FGF21 levels during the trial were increased both in the fructose and water groups, whereas the changes in the values between the groups were not significantly different in the RCT (*p* = 0.505). There was no difference in dietary consumption and physical activity between the groups. BAT activity at baseline and after cold exposure was not increased by daily fructose consumption and was not different between the fructose and water groups. For cold exposure, there was no significant difference in shivering response, cold sensation, and discomfort between the two groups (data not shown).

**Table 2:** Changes in the variables during 2-week fructose consumption in the randomized controlled trial

	Fructose (*n* = 11)			Control (*n* = 10)			*p*
	Pre	Post	⊿	Pre	Post	⊿	
Age (year)	22.1±1.4			21.7±1.6			0.568
Height (cm)	175.9±5.8			171.0±5.3			0.056
Weight (kg)	63.8±7.2	63.6±6.6	− 0.3±0.8	60.6±6.1	60.5±6.1	−0.1±0.5	0.591
BMI (kg/m^2^)	20.6±2.1	20.5±2.0	− 0.1±0.3	20.7±1.8	20.7±1.9	−0.1±0.2	0.736
Body fat (%)	14.6±3.8	15.3±3.1	0.7±1.2	15.1±3.3	15.8±4.0	0.7±1.1	0.926
Blood metabolic parameters							
Glucose (mg/dL)	98.0±8.5	93.4±6.0	− 4.6±9.3	90.9±4.4	91.7±7.8	0.9±8.4	0.173
FFA (mEq/L)	0.24±0.13	0.20±0.13	− 0.04±0.19	0.27±0.08	0.29±0.27	0.02±0.28	0.835*
Triglyceride (mg/dL)	63.0±23.0	83.0±58.0	20.0±59.0	58.0±26.0	59.0±21.0	1.0±27.0	0.505*
Total cholesterol (mg/dL)	165.0±28.0	168.0±31.0	3.0±10.0	167.0±23.0	167.0±27.0	−1.0±13.0	0.477
HDL cholesterol (mg/dL)	60.0±11.0	59.0±9.0	0.0±6.0	56.0±9.0	54.0±10.0	−2.0±4.0	0.256*
LDL cholesterol (mg/dL)	92.0±24.0	90.0±22.0	− 1.0±8.0	100.0±24.0	98.0±22.0	2.0±14.0	0.884
FGF21(pg/mL)	125±74	182±174	57±144	158±59	227±183	68±169	0.505*
Dietary consumption (/day)							
Energy (kcal)	1914±524	1726±647	− 188.0±589.7	1433±415	1492±518	58.4±310.8	0.253
Protein (g)	63.8±20.7	59.6±25.5	− 4.1±17.7	49.7±15.2	53.4±20.3	3.7±12.9	0.267
Fat (g)	68.6±20.6	63.6±27.0	− 4.9±18.0	51.9±15.9	54.4±18.0	2.4±9.4	0.262
Carbohydrate (g)	239.4±72.3	206.6±78.2	− 32.8±101.0	180.2±56.0	185.4±70.0	5.2±56.6	0.308
PA (METs-h/week)	51.9±36.9	67.8±54.7	15.8±45.4	59.7±69.5	76.8±122	17.1±58.5	0.123*
BAT activity (°C)							
Baseline	0.49±0.22	0.55±0.24	0.06±0.14	0.47±0.21	0.47±0.13	0.00±0.19	0.433
30-min cold exposure	0.87±0.43	0.86±0.35	− 0.02±0.21	0.78±0.32	0.70±0.35	−0.08±0.26	0.536
60-min cold exposure	0.93±0.42	0.92±0.40	− 0.01±0.11	0.93±0.25	0.76±0.36	−0.17±0.31	0.128
Maximal activity	1.00±0.40	1.00±0.34	0.00±0.09	0.94±0.25	0.87±0.32	−0.07±0.23	0.366

Student’s *t* test (for normally distributed data) or Mann-Whitney *U* test (for non-normally distributed data) were performed

*BAT* brown adipose tissue, *BMI* body mass index, *FFA* free fatty acid, *FGF21* fibroblast growth factor 21, *HDL* high-density lipoprotein, *LDL* low-density lipoprotein, *MET* metabolic equivalent, *PA* physical activity

*Results of the Mann-Whitney *U* test

### Associated factors of BAT activity in the RCT

**Fig. 4:**
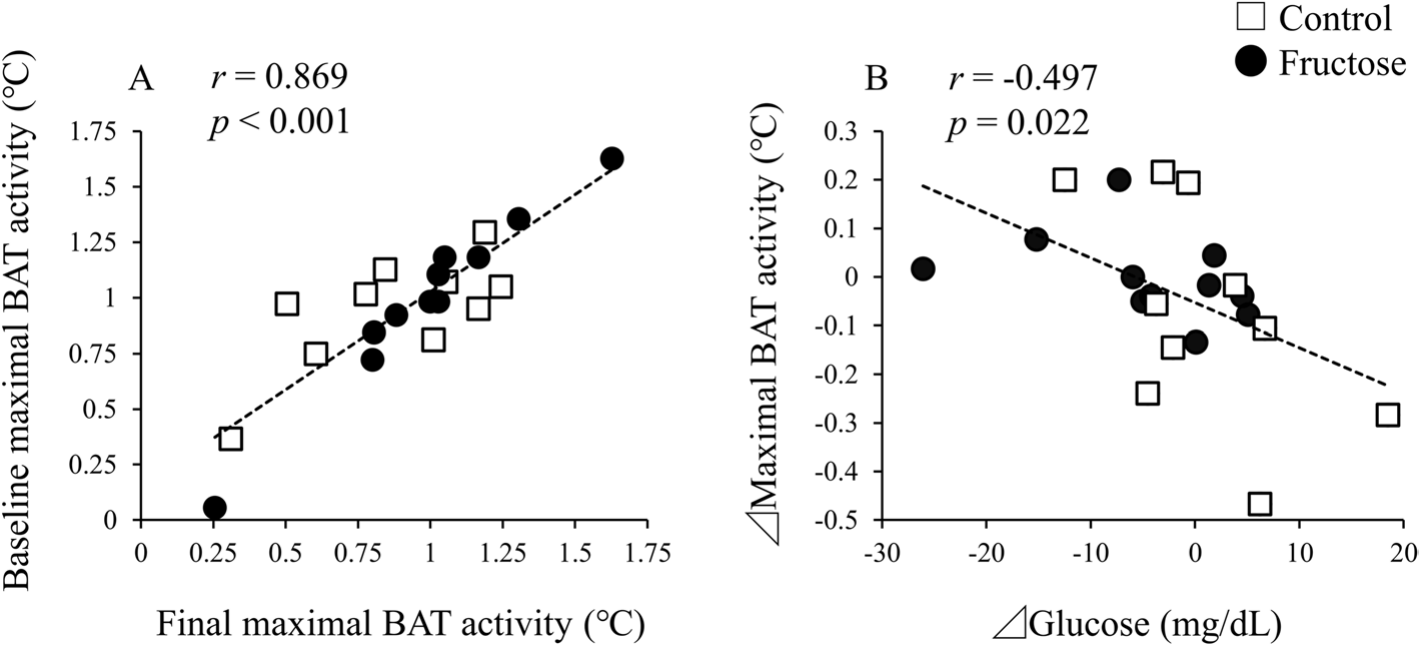
Correlation analyses in the RCT. **A** Correlation of maximal BAT activity between the baseline and final measurements. **B** Correlation between changes in maximal BAT activity and plasma glucose levels. Open square shows the control group, and closed circle shows the fructose group. Results of Pearson’s correlation coefficients are shown. BAT, brown adipose tissue; RCT, randomized controlled trial

Correlation analyses were performed to determine the associated factors of BAT activity changes during the 2-week intervention (Table 3). As shown in Fig. 4A, BAT activity at the baseline and the final measurements were strongly and positively correlated in the RCT (*r* = 0.869, *p* < 0.001). The changes in BAT activity during the trial were not associated with changes in plasma FGF21 levels (*r* = − 0.065, *p* = 0.780), whereas the changes in maximal BAT activity were significantly and negatively correlated with changes in plasma glucose levels during the 2-week intervention (Fig. 4B; *r* = −0.497, *p* = 0.022). Changes in anthropometric values, lipid metabolic parameters, dietary consumption, and physical activity were not associated with the changes in BAT activity.

**Table 3:** Correlations between changes in BAT activity and other variables in the randomized control trial (*n* = 21)

Dependent variable	⊿Maximal BAT activity (°C)	
	*r*	*p*
⊿BMI (kg/m^2^)	− 0.053	0.819
⊿Body fat (%)	− 0.086	0.710
Blood metabolic parameters		
⊿Glucose (mg/dL)	− **0.497**	**0.022**
⊿FFA (mEq/L)	− 0.224	0.329
⊿Triglyceride (mg/dL)	0.009	0.970
⊿Total cholesterol (mg/dL)	0.128	0.579
⊿HDL cholesterol (mg/dL)	0.062	0.791
⊿LDL cholesterol (mg/dL)	0.109	0.639
⊿FGF21(pg/mL)	− 0.065	0.780
Dietary consumption (/day)		
⊿Energy (kcal)	0.128	0.580
⊿Protein (g)	0.260	0.256
⊿Fat (g)	0.166	0.473
⊿Carbohydrate (g)	0.082	0.724
⊿PA (METs-h/week)	− 0.368	0.101

The results of Pearson’s correlation coefficients are shown

*BAT* brown adipose tissue, *BMI* body mass index, *FFA* free fatty acid, *FGF21* fibroblast growth factor 21, *HDL* high-density lipoprotein, *LDL* low-density lipoprotein, *MET* metabolic equivalent, *PA* physical activity

Boldface indicates significance (*p* < 0.05)

## Discussion

We performed a randomized cross-over trial and an RCT to evaluate whether dietary-induced FGF21 secretion activates BAT thermogenesis. In the present study, oral fructose load increased plasma FGF21 levels more than 2-fold but did not influence BAT activity in both the acute and short-term interventions. The temporary increase in circulating FGF21 level may have little or no effect on BAT thermogenesis in young healthy adults.

The thermogenic action and mechanism of FGF21 have been explored using transgenic and high-dose models in rodents [16, 29], which showed abnormal levels of circulating FGF21. Because recombinant human FGF21 has a short half-life of less than 2 h in mice and primates [30], the pharmacological properties in clinical trials were examined using FGF21 analogs with a prolonged half-life [17, 18]. Unlike the above studies, the present study found that the increased FGF21 levels 2 h after oral fructose load were decreased within 30 min. Therefore, the weak and short actions of FGF21 were considered as the reason why the fructose intake did not activate BAT thermogenesis. It was reported that BAT activity is increased by methods of sympathetic nervous system activation such as cold exposure [5, 6] and oral intake of food ingredients [4, 11–13]. At this point, these methods have more advantages than FGF21 induction regarding BAT activation. Moreover, it was reported that a hypercaloric fructose diet was associated with risks of obesity [31] and fatty liver [32]. Both obesity and fatty liver accumulation are independent factors of chronic high FGF21 levels [33, 34], which is considered an FGF21-resistant state [35] and related to the onset of type 2 diabetes and cardiovascular diseases [36–39]. Thus, the present study supports these previous studies that found that fructose intake has health disadvantages.

The present study found a dose-response relationship between 30 g oral fructose and blood FGF21 levels, which supports the findings of a previous study in the USA [23]. This suggests that genetic and lifestyle background do not affect oral fructose-induced FGF21 secretion and that the oral amount of fructose was adequate to increase circulating FGF21 levels. On the other hand, we found a significant decrease in plasma FGF21 levels after drinking water in the single-load trial. This finding may be explained by a diurnal rhythm of circulating FGF21, which decreases from morning to afternoon [40]. Daily fructose intake at breakfast may influence the diurnal rhythm of FGF21, although the physiological role requires further investigation. Circulating FFA levels were decreased around 2 h after oral fructose load as seen in this and a previous study [41]. Fructose consumption activates hepatic ChREBP [24], which induces triglyceride synthesis in the liver [42]. It is likely that hepatic FFA uptake increased due to lipogenesis, and a temporary decrease in FFA levels was observed. Previous studies reported that FGF21 administration induced glucose uptake in both adipocyte and adipose tissue [16, 43]; however, in the present study, a single load did not show changes in plasma glucose levels during increased plasma FGF21. Our findings suggest that an acute increase in endogenous FGF21 may not have an impact on whole glucose homeostasis in healthy young men.

The present study found that both anthropometric and blood metabolic parameters did not significantly change after 2 weeks of daily consumption of 30 g of fructose. Our findings suggest that short-term fructose consumption does not have both beneficial and adverse effects on the parameters of healthy young adults. Daily fructose consumption did not change baseline FGF21 levels; thus, daily FGF21 secretion may not modulate the diurnal rhythm of FGF21. Previous studies reported that resting levels of circulating FGF21 were increased by a low protein diet [44] and were decreased by endurance exercise [45, 46]. We assessed macronutrient intakes and physical activity using standardized questionnaires; however, these associated factors were unchanged between the baseline and final measurements. For the correlation analyses, a highly reproducible correlation was observed between the baseline and final measurements of BAT activity in the RCT. Our findings suggest that BAT activity was stable within individuals throughout the study period and was not influenced by the intervention. In addition, changes in glucose levels were only negatively associated with BAT activity in young men. The role in glucose disposal of activated BAT has been reported [4–6]; thus, unlike the consumption of fructose, changes, such as environmental and/or daily variation in BAT activity, may be associated with glucose disposal and negative correlation in the RCT.

Body temperature is controlled by shivering thermogenesis in skeletal muscle and non-shivering thermogenesis in BAT [47]. Cold-induced shivering is limited in neonates due to underdeveloped skeletal muscle. As a result, human neonates have various BAT sites including the interscapular, neck, and mediastinum regions [47]. Although BAT has a role in acclimatization to cold exposure after adolescence, the presence and volume of BAT decline with age [47]. The underlying factors of BAT adaptability are unclear. Previous studies reported that FGF21-deficient mice displayed an impaired response to cold stress [48], and the diurnal reduction of FGF21 was suppressed by cold exposure in healthy adults [49, 50]. Thus, FGF21 may be a mediator of cold-induced thermogenesis in adults; however, the present study did not find either the suppressed FGF21 reduction by cold exposure or the association of plasma FGF21 level with BAT activity in young men. Because female subjects participated in these previous studies [49, 50], our findings may be partly explained by sex differences in FGF21 responsiveness and/or BAT thermogenesis in cold adaptation.

This study has several limitations. The sample size was relatively small. Blinding was not performed because fructose has a sweet taste. Young healthy subjects participated; therefore, our findings may not be generalizable to other age categories and patients with metabolic diseases. BAT activity was not assessed by positron emission tomography-computerized tomography, which is the gold standard method for evaluating BAT [2]. In the cross-over trial, cold-induced changes in diurnal FGF21 rhythm should be considered [49, 50]. Although dietary-induced FGF21 secretion could not activate BAT, the effects of longer FGF21 action, such as using an FGF21 analog, on thermogenesis were unclear. Further clinical research is necessary to explore the physiological role of FGF21 in human BAT.

## Conclusions

Oral fructose load induces a temporary increase in circulating FGF21 levels; however, the increased FGF21 does not activate BAT thermogenesis in healthy young men. Although it tends to evaluate the increase and/or decrease levels in the hormone, the implications of endogenous FGF21 on physiological function require further exploration.

## Data Availability

The datasets used and/or analyzed during the present study are available from the corresponding author on reasonable request.

## Abbreviations

BAT: Brown adipose tissue
ChREBP: Carbohydrate response element binding protein
FGF21: Fibroblast growth factor 21
RCT: Randomized controlled trial
